# High-Frequency Fluctuations in Post-stenotic Patient Specific Carotid Stenosis Fluid Dynamics: A Computational Fluid Dynamics Strategy Study

**DOI:** 10.1007/s13239-019-00410-9

**Published:** 2019-04-01

**Authors:** Viviana Mancini, Aslak W. Bergersen, Jan Vierendeels, Patrick Segers, Kristian Valen-Sendstad

**Affiliations:** 1 IBiTech-bioMMeda, Ghent University, 9000 Ghent, Belgium; 2 Department of Computational Physiology, Simula Research Laboratory, 1364 Fornebu, Norway; 3 Department of Flow Heat and Combustion Mechanics, Ghent University, 9000 Ghent, Belgium

**Keywords:** CFD, Finite elements, Carotid stenosis, Oasis, LES

## Abstract

**Purpose:**

Screening of asymptomatic carotid stenoses is performed by auscultation of the carotid bruit, but the sensitivity is poor. Instead, it has been suggested to detect carotid bruit as neck’s skin vibrations. We here take a first step towards a computational fluid dynamics proof-of-concept study, and investigate the robustness of our numerical approach for capturing high-frequent fluctuations in the post-stenotic flow. The aim was to find an ideal solution strategy from a pragmatic point of view, balancing accuracy with computational cost comparing an under-resolved direct numerical simulation (DNS) approach vs. three common large eddy simulation (LES) models (static/dynamic Smagorinsky and Sigma).

**Method:**

We found a reference solution by performing a spatial and temporal refinement study of a stenosed carotid bifurcation with constant flow rate. The reference solution $\left( {\Delta x = 1.92 \times 10^{ - 4} \;{\text{m}},\; \Delta t = 5 \times 10^{ - 5} \;{\text{s}}} \right)$ was compared against LES for both a constant and pulsatile flow.

**Results:**

Only the Sigma and Dynamic Smagorinsky models were able to replicate the flow field of the reference solution for a pulsatile simulation, however the computational cost of the Sigma model was lower. However, none of the sub-grid scale models were able to replicate the high-frequent flow in the peak-systolic constant flow rate simulations, which had a higher mean Reynolds number.

**Conclusions:**

The Sigma model was the best combination between accuracy and cost for simulating the pulsatile post-stenotic flow field, whereas for the constant flow rate, the under-resolved DNS approach was better. These results can be used as a reference for future studies investigating high-frequent flow features.

## Introduction

Carotid stenosis is a progressive and local buildup of plaque in the carotid bifurcation, leading to a local narrowing of the lumen. The major risk consists of plaque rupture with subsequent debris and thrombi being transported downstream where they can cause a blockage leading to a stroke and consequent neurologic deficits.^35^ Asymptomatic carotid artery stenoses (ACAS), which affects 1.6% of the population,^8^ are rarely detected unless diagnosed with another associated cardiovascular disease.^42^

A characteristic feature of stenoses is that the downstream flow is turbulent-like, with high-frequent pressure fluctuations.^3^ These fluctuations can traverse the soft neck tissue as mechanical waves, and present as a bruit or skin vibration. The stenosis-induced turbulent-like flow is therefore a strong marker for inferring the presence of a stenosis.

The current clinical practice for ACAS screening is auscultation of the carotid bruit.^32^ Auscultation is only applied if the physician suspects presence of a stenosis, i.e., if correlated risk factors are present,^42^ and is operator-dependent, with low sensitivity^23^ due to the presence of background noise.^37^ Carotid auscultation is hence not sufficient to infer the presence of a stenosis, whose diagnosis hence requires confirmation by techniques which are usually not available to a general practitioner, i.e., ultrasound or tonometry.

To overcome the abovementioned challenges, the CARDIS project proposes to instead infer the presence of stenosis by measuring skin vibrations using a newly developed multi-beam laser Doppler vibrometry device,^21^ with increased temporal resolution, with 10 μs at a sample rate of 100 kHz, and reduced noise level, with less than 1 *μ*m for 5 s time measurement in the 1–1000 Hz range.^22^ The device has already proven suitable for measuring physiological signals from skin movements, such as pulse wave velocity and heart rate.^7^, ^38^ The new device could allow rapid and consistent non-contact screening for ACAS, and thus detection prior to a traumatic event. As part of this project, we combine *in vitro* experiments and computational fluid dynamics (CFD) flow simulations to show a ‘theoretical’ proof of concept of the device before clinical testing. However, the efficacy of CFD relies on the robustness of the numerical methods and their ability to reproduce experimental results, and has proven challenging, especially for transitional flows.^4^ In particular, the use of numerical schemes, such as first order UPWIND schemes, well-known to be dissipative,^34^ is common in the biomedical literature.^46^ The choice of the correct numerical methodology is hence crucial for reliable simulations.

We have previously used an under-resolved DNS approach with rigid walls for two biomedical benchmarks^4^, ^16^ and biomedical applications such as aneurysms^15^, ^44^, ^45^ and vascular junctions.^28^ The first aim of this study was to find an adequate under-resolved DNS solution from a spatial and temporal refinement study with respect to the smallest scales, and from a pragmatic biomedical engineering point of view balancing the computational cost with accuracy.

Directly calculating the smallest scales of the turbulent-like flow requires large computational resources, and is therefore not routinely performed in the biomedical literature. However, we also know that the smallest scales have little energy, and only contribute to dissipation. We can therefore model these scales, for instance by means of large eddy simulations (LES). Applying LES can allow for the use of a coarser grid, since we can model the scales we do not capture, referred to as the sub-grid scales (SGS). LES simulations depend on the properties of the SGS model, for instance its ability to mimic the near-wall behavior. The second aim of this study was therefore to assess whether three commonly used SGS models (Smagorinsky, Sigma and Dynamic Smagorinsky) are able to replicate our reference solution for both constant—at peak systole—and pulsatile flow rates on a coarser mesh, and thus reducing computational cost without significant loss of resolution of the high-frequent flow features. The study was performed in an anatomically correct model geometry, retrieved from a patient with significant carotid stenosis, subjected to physiologically relevant boundary conditions.

## Methods

Computed tomography angiography images of a common carotid bifurcation with severe stenosis (76% narrowing computed by means of the *NASCET* method^36^) in the internal carotid artery (ICA) were obtained from a 75 years old male patient, who gave informed consent for the use and further processing of the images.

**Figure 1:**
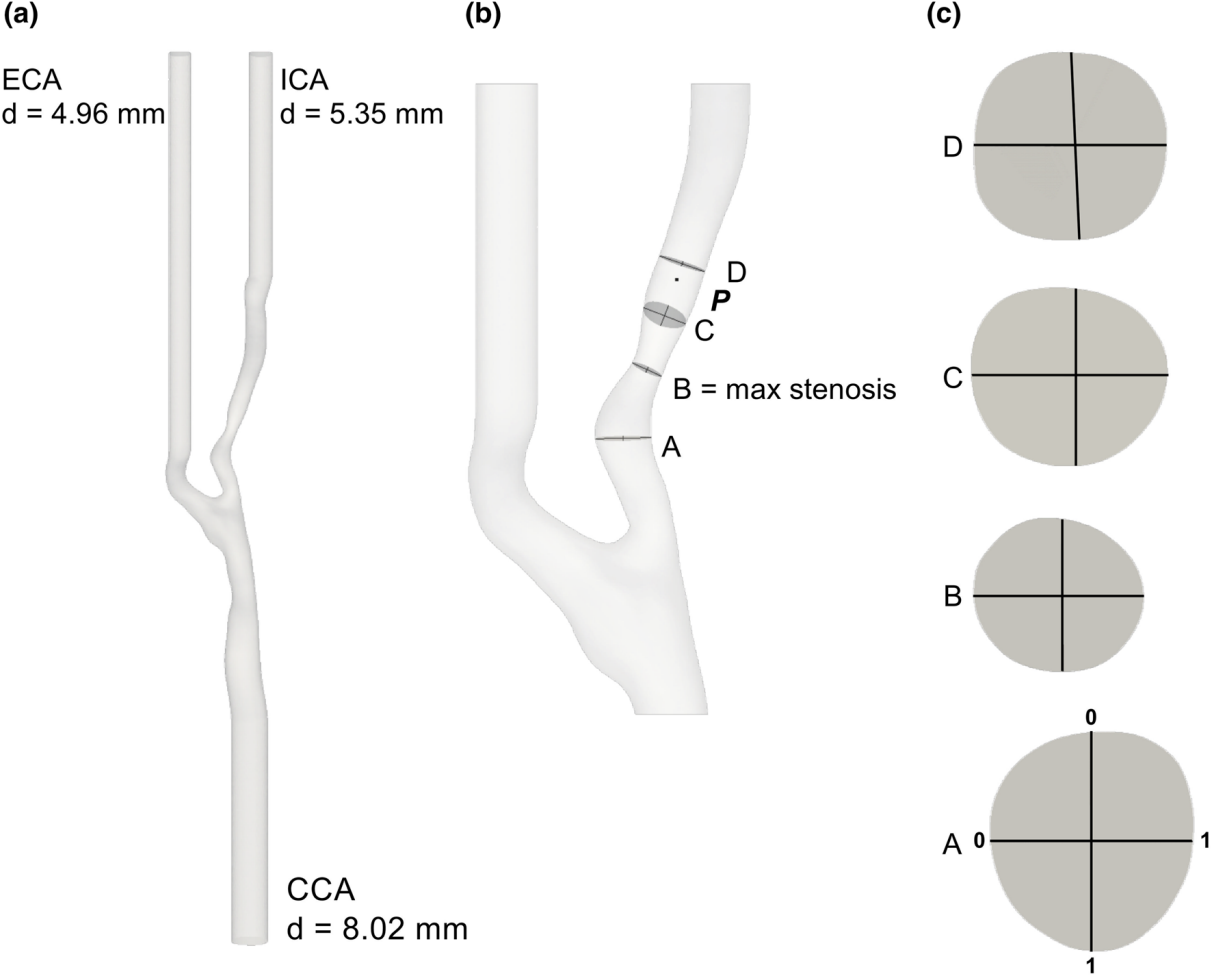
(a) Patient-specific geometry with flow extensions, where ‘d’ indicates the diameters of CCA, ECA, and ICA. (b) The point **P**, the slices A, B, C, and D and two perpendicular lines on each slice, with a detail of each slide in (c), indicate where the velocity and pressure were sampled.

The medical images were segmented using 3D Slicer^13^ to obtain an anatomically plausible model of the vasculature, and the inlets—the common carotid artery (CCA)—and outlets—the ICA and the external carotid artery (ECA)—were extruded using PyFormex^11^ to ensure that the flow was fully developed. Depicted in Fig. 1 are the full computational domain (a), and the region of interest (b). The relevant fields were evaluated in point **P**, on four slices (A, B, C and D), two orthogonal lines per slice (Fig. 1c), and points along the centerline. Point **P** was located 8 mm, 1 diameter (d), downstream of the stenosis, and the slices A, B, C, and D were located − 0.75 d, 0.0 d, 0.6 d, and 1.2 d, relative to the center of the stenosis, respectively.

We used the Vascular Modelling Toolkit 30 to create five meshes with a local refinement in the stenosed and downstream region, with the same size ratio between the coarse and fine region for all meshes. The resulting meshes had 200 thousand (K), 2 million (M), 6 M, 22 M, and 50 M tetrahedral elements, now referred to as the 200K, 2M, 6M, 22M, and 50M meshes, respectively. Mesh details are listed in Table 1. An additional mesh with 11 million elements (11M) was created similarly in order to allow a preliminary common simulation, as better explained in the next paragraphs.

**Table 1:** Mesh characteristics.

Mesh name	Number elements (–)	Average cell length $(\Delta x$) (m)	Number of boundary layers (–)
200K	$2 \times 10^{5}$	$9.13 \times 10^{ - 4}$	1
2M	$2 \times 10^{6}$	$4.63 \times 10^{ - 4}$	4
6M	$6 \times 10^{6}$	$3.04 \times 10^{ - 4}$	4
22M	$22 \times 10^{6}$	$1.92 \times 10^{ - 4}$	4
50M	$50 \times 10^{6}$	$1.44 \times 10^{ - 4}$	4

**Figure 2:**
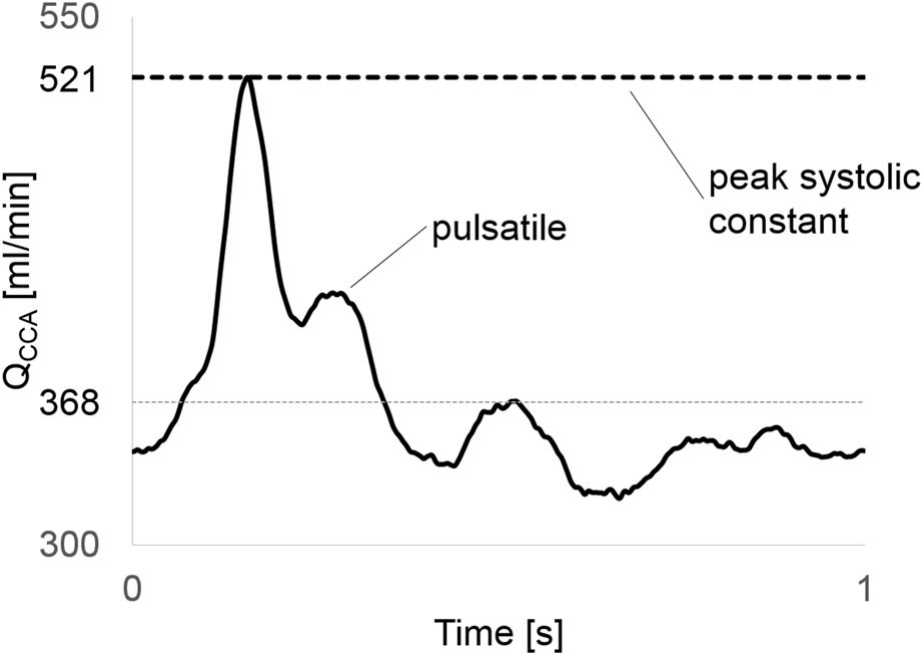
A typical carotid artery waveform (average 368 mL/min, peak 521 mL/min) was used in this study. Constant flow rate equivalent with peak systolic flow was set as inlet flow for the refinement study in order to allow a more rigorous assessment of grids resolution.

The fluid properties were set to mimic water, a Newtonian fluid with kinematic viscosity of $\nu = 1 \times 10^{ - 6}$ m^2^/s to ease future comparison to *in vitro* experiments. We applied two types of inlet conditions; a parabolic profile with constant flow rate of 520 mL/min leading to a Reynolds number (*Re*) of 1380, and a pulsatile Womersley flow (averaged flow rate of 370 ml/min) with period of 1 s and an averaged *Re* = 980,^9^ see Fig. 2. The former is to enable rigorous worst-case assessment of the smallest temporal and spatial scales in the flow, although at the cost of losing the pulsatility in a physiological flow condition.

We assumed rigid walls, prescribed a no-slip boundary condition along the vessel walls, and enforced a flow split of 43.8% in the ICA/CCA, based on the model presented in Groen *et al*.^10^ by prescribing a Womersley profile on the ECA outlet and zero pressure at the ICA outlet.

To cheaply washout initial transients associated with the artificial initial conditions we computed the flow at the 11M mesh for 2 physical seconds, equivalent of one and a half flow-throughs with model length of 0.2 m and peak inlet velocity of 0.15 m/s. We projected the last time step of the solution onto each mesh as an initial condition.

Simulations were performed using the open-source verified^27^ and validated^4^ finite element CFD solver *Oasis,*^27^ where special care was taken to ensure a kinetic energy-preserving and minimally-dissipative numerical solution of the Navier–Stokes equation. We used linear Lagrange elements (${\mathbb{P}}_{1} - {\mathbb{P}}_{1}$) for both velocity and pressure.

The spatial refinement study was simulated with $\Delta t = 5 \times 10^{ - 5}$ s, while the temporal refinement study was performed on the least computationally expensive mesh which gave adequate results with varying time step: $\Delta t = 1 \times 10^{ - 4} , \;5 \times 10^{ - 5} , \;1 \times 10^{ - 5}$ and $5 \times 10^{ - 6}$ seconds. The pair of $\Delta t$ and mesh size that provided the best tradeoff between computational cost, and accuracy of resolving high-frequent flow features was used as the reference solution for comparison with the LES simulations.

A generic form of functional SGS models can be described as in Eq. (1), whereas the SGS viscosity ($\nu_{SGS}$) is defined in Eq. (2).

1 $$\tau_{ij}^{SGS} - \frac{1}{3}\tau_{kk}^{SGS} \delta_{ij} = 2\rho \nu_{SGS} \left( {S_{ij} - \frac{1}{3}S_{kk} \delta_{ij} } \right)$$

2 $$\nu_{SGS} = \left( {C_{m} \Delta } \right)^{2} \times D_{m} (\varvec{u})$$

The SGS tensor $$\tau_{ij}^{SGS}$$ depends on the strain tensor of the resolved scaled $$S_{ij}$$ and on the subgrid-scale viscosity $$\nu_{SGS}$$, which is a function of the cut-off length scale $$\Delta$$ and of two model-specific parameters $$D_{m}$$ and $$C_{m} .$$$$D_{m}$$ is the model specific differential operator related to the resolved velocity field $$\varvec{u}$$ and it sets the properties of the model, for instance, the near wall behavior. $$C_{m}$$ is the model constant and it sets the amount of energy drained from the resolved scales.

We applied three different SGS models: the static Smagorinsky model^39^ with $C_{m} = 0.168$, the Sigma model^29^ with $C_{m} = 1.5$, and the dynamic Smagorinsky model, for which the model-specific parameters were updated every time step following Meneveau *et al*.^26^ The LES simulations were performed on the mesh with one spatial refinement level lower, i.e., on a coarser mesh, relative to the reference solution, but with the same $\Delta t$ to ensure a fair comparison.

The number of cores, reported in Table 2, was kept constant for each mesh, regardless of time step or SGS model. The workload of each simulation was obtained as number of cells divided by number of cores. All simulations were stopped after 5 physical seconds and flow statistics were based on the last four seconds, as one physical second was quantitatively found to be sufficient for washing out the artifacts from the initial condition. A list of all simulations can be found in Table 3.

**Table 2:** Number of cores and workload per core for each simulation.

	Number of cells	Number of cores	Workload	Workload/6M workload
200K	$174.37 \times 10^{3}$	16	$1.09 \times 10^{4}$	0.06
2M	$1.84 \times 10^{6}$	16	$1.15 \times 10^{5}$	0.62
6M	$5.97 \times 10^{6}$	32	$1.87 \times 10^{5}$	1.00
22M	$22.43 \times 10^{6}$	96	$2.34 \times 10^{5}$	1.25
50M	$51.14 \times 10^{6}$	128	$4.00 \times 10^{5}$	2.14

The number of nodes was kept constant for each mesh. There were 16 cores in each node

**Table 3:** List of the simulations performed for the three studies reported in this paper, with details on the mesh size, time step size, inlet type, end time and LES model.

Study	Mesh size	Time step size $(\Delta t$) [s]	Inlet type	LES model
Spatial refinement	**200K**	$5 \times 10^{ - 5}$	Constant	None
	**2M**	$5 \times 10^{ - 5}$	Constant	None
	**6M**	$5 \times 10^{ - 5}$	Constant	None
	**22M**	$5 \times 10^{ - 5}$	Constant	None
	**50M**	$5 \times 10^{ - 5}$	Constant	None
Temporal refinement	22M	$\bf{1 \times 10^{ - 4}}$	Constant	None
	22M	$\bf{5 \times 10^{ - 5}}$	Constant	None
	22M	$\bf{1 \times 10^{ - 5}}$	Constant	None
	22M	$\bf{5 \times 10^{ - 6}}$	Constant	None
LES	6M	$5 \times 10^{ - 5}$	Constant	**Smagorinsky**
	6M	$5 \times 10^{ - 5}$	Constant	**Dynamic Smagorinsky**
	6M	$5 \times 10^{ - 5}$	Constant	**Sigma**
	6M	$5 \times 10^{ - 5}$	Pulsatile	**Smagorinsky**
	6M	$5 \times 10^{ - 5}$	Pulsatile	**Dynamic Smagorinsky**
	6M	$5 \times 10^{ - 5}$	Pulsatile	**Sigma**
Reference solution	6M	$5 \times 10^{ - 5}$	Pulsatile	None
	22M	$5 \times 10^{ - 5}$	Pulsatile	None

The text in bold highlights the parameters changed in each category

For visual inspection of the coherent vortical structures in the turbulent-like post-stenotic flow we computed the Q-criterion as in Eq. (3), which is a spatial region where the Euclidean norm of the vorticity tensor $\vec{\varOmega }$ dominates the strain rate tensor $\vec{S}$.^12^

3 $$Q = \frac{1}{2} \left[ {\left| {\vec{\varOmega }} \right|^{2} - \left| {\vec{S}} \right|^{2} } \right] > 0 $$

Reynolds decomposition was used for all constant flow rate simulations to separate the instantaneous velocity, $\varvec{u}(\varvec{x}, t)$, from the time averaged, $\bar{\varvec{u}}(\varvec{x})$, and fluctuating, $\varvec{u^{\prime}}(\varvec{x}, t)$, components, i.e., $\varvec{u} = \bar{\varvec{u}} + \varvec{u^{\prime}}.$ Taking the fluctuating velocity magnitude signal, $|\varvec{u^{\prime}}|$, as input we computed the power spectral density (PSD) using Welch’s method^12^ with 16 segments and a Hanning windowing function with 50% overlap. The turbulent kinetic energy ($tke$) was calculated as $tke = \frac{1}{2}\mathop \sum \limits_{i = 1}^{3} \overline{{\varvec{u^{\prime}}({x}_{{i}} , t)^{2} }}$ where $\varvec{u^{\prime}}(x_{i = 1:3} , t)$ are the components of the fluctuating velocity.

**Figure 3:**
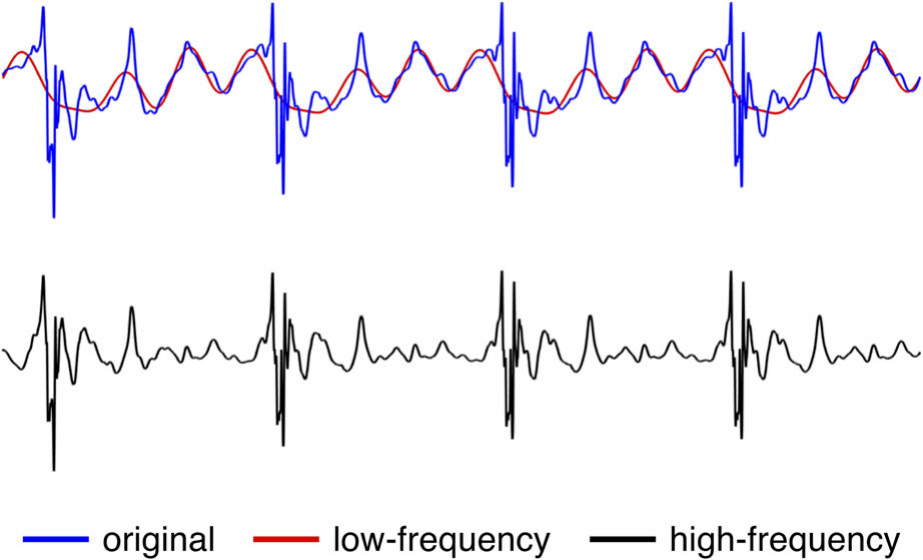
The original velocity signal was decomposed in 1000 harmonics and its low-frequency components were separated from the high-frequency components by means of a 16^th^ mode threshold.

For pulsatile simulations, however, the Reynolds decomposition was no longer directly applicable as it was not possible to obtain a mean flow without simulating tens of cardiac cycles to obtain a phase averaged mean. We therefore applied a high pass filter, like suggested by Khan *et al.*,^14^ to the original pulsatile velocity $\varvec{u}\left( {x,t} \right)$ to obtain a good approximation for $|\varvec{u^{\prime}}|$. We created a fast Fourier decomposition with 1000 components of the velocity signal, and we set the first 16 modes, representing the low frequencies, to 0, see Fig. 3 for a visual example.

## Results

### General Flow Features

**Figure 4:**
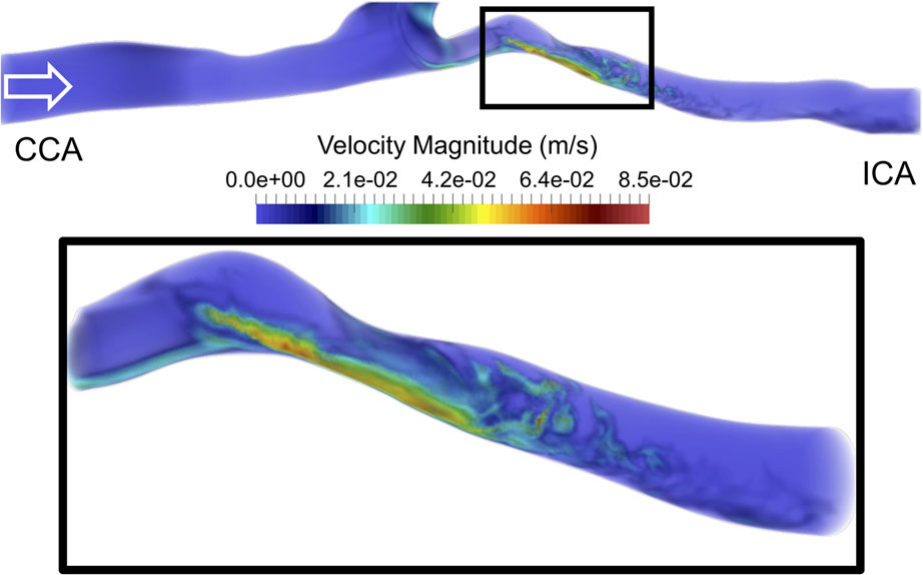
Top panel: volumetric rendering of the instantaneous velocity magnitude in the common and internal carotid arteries. Bottom panel: enlargement of the box in the top panel, focusing on the stenosis and downstream region. The flow became perturbed already before reaching the stenosis. The flow then accelerates in the stenosis and decelerates once out, causing the jet to break down into turbulent-like structures.

We first focused on the instantaneous flow features at $t = 2.0$ s obtained on the 22M-element mesh with $\Delta t = 5 \times 10^{ - 5}$ s and a constant flow rate (Fig. 4). The top of Fig. 4 shows that the flow in the CCA was stable until the carotid bifurcation, while vortical structures formed at the bifurcation. Moreover, because of the non-parabolic flow entering the ICA and pronounced curvature, the flow became unstable already upstream of the stenosis, as observed in the left most section of the zoomed in box, see Fig. 4—bottom panel. The flow accelerated through the stenosis before the jet broke down into an unstable flow downstream of the stenosis. The flow instabilities quickly dissipated further downstream, and the flow relaminarized.

### Sensitivity Analysis

#### Spatial Refinement Study

**Figure 5:**
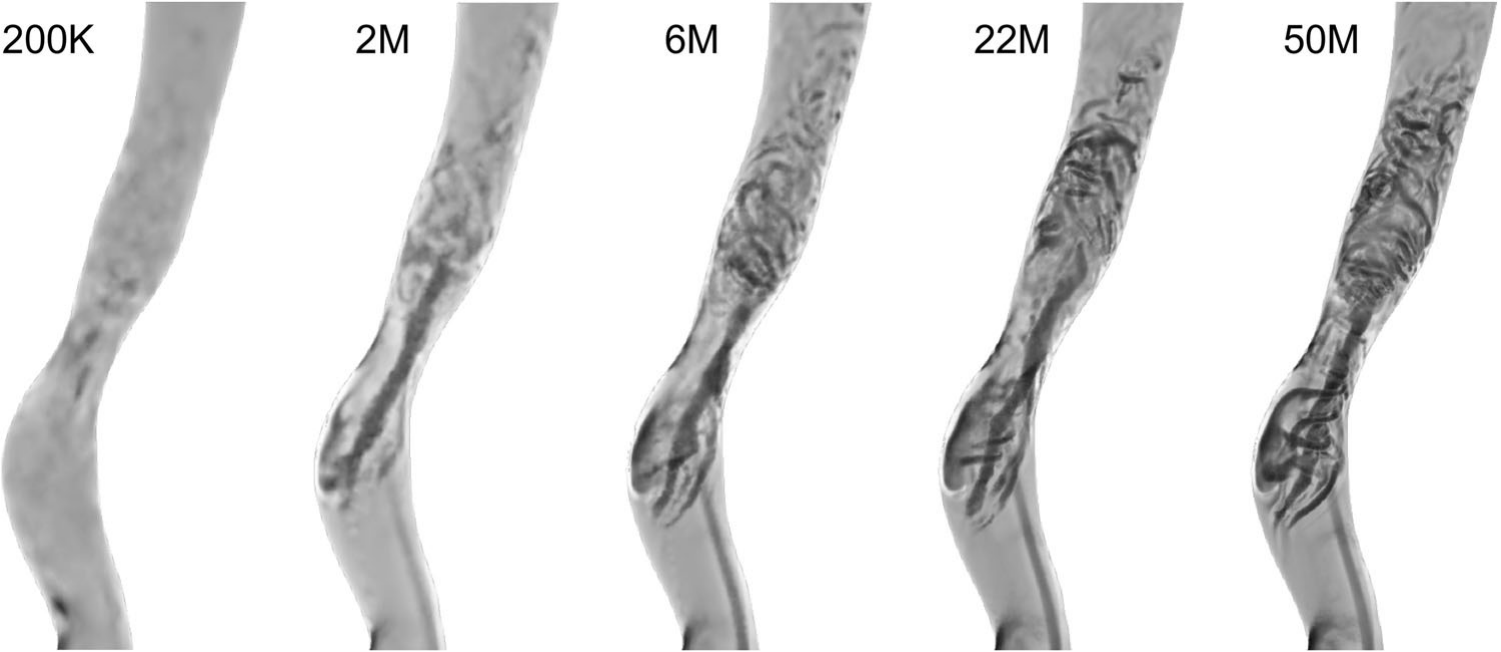
The vortical structures in the ICA for the 200K mesh are almost entirely missing. While the 2M and 6M meshes already show some turbulent-like flow features, it is only on the 22M and 50M that these vortical structures are clearly distinct.

We first performed a qualitative assessment of the spatial refinement study based on the Q-criterion of instantaneous velocity fields in the ICA at identical times points ($t = 2.0$ s). In Fig. 5, from left to right with increasing mesh resolution, one can observe a consistent increase in the number of vortices. In the 2M and 6M meshes, it is possible to observe some larger coherent vortical structures, while in the 200K mesh these were almost entirely absent. However, the 22M and 50M element simulations showed smaller and more complex structures and were hence phenotypically different from the 2M and 6M. The vortices of the downstream region were visually easier to see in the 50M than in the 22M, however one can observe the same type and distribution of vortices, showing similarities between the 22M and 50M meshes.

**Figure 6:**
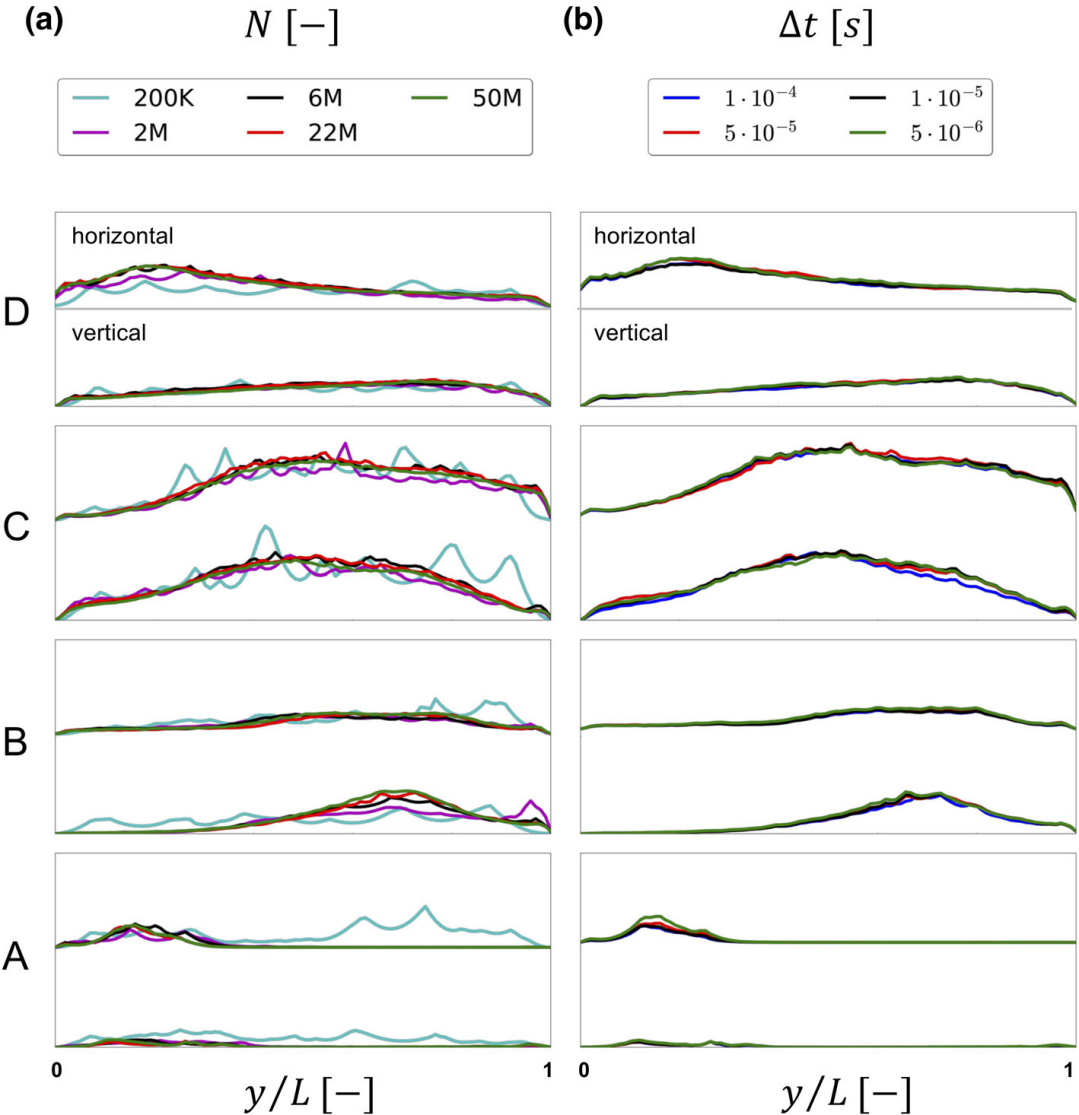
Time-averaged $tke$ over vertical and horizontal lines on slices A to D for the five meshes in a) and for the four time steps in b).

To further assess the results of the spatial refinement study, we considered the time-averaged $tke$ obtained by Reynolds decomposition of the velocity across two perpendicular lines on each of the four slices A–D, previously shown in Fig. 1c. The left panel of Fig. 6 depicts the $tke$ values along the vertical (top to bottom) and horizontal (left to right) lines for each slice. In all lines, both upstream and downstream of the stenosis, we observed that while the 200K-element simulation was alike any other simulation, the 2M and 6M ones were similar, but relatively different from the 22 and 50M simulations. Of note is also that even though the spatial resolution of 200K element simulation was too coarse to capture any vortex structures in Fig. 5, the flow is still turbulent-like with high-frequent fluctuations, as evident in Fig. 6a.

**Figure 7:**
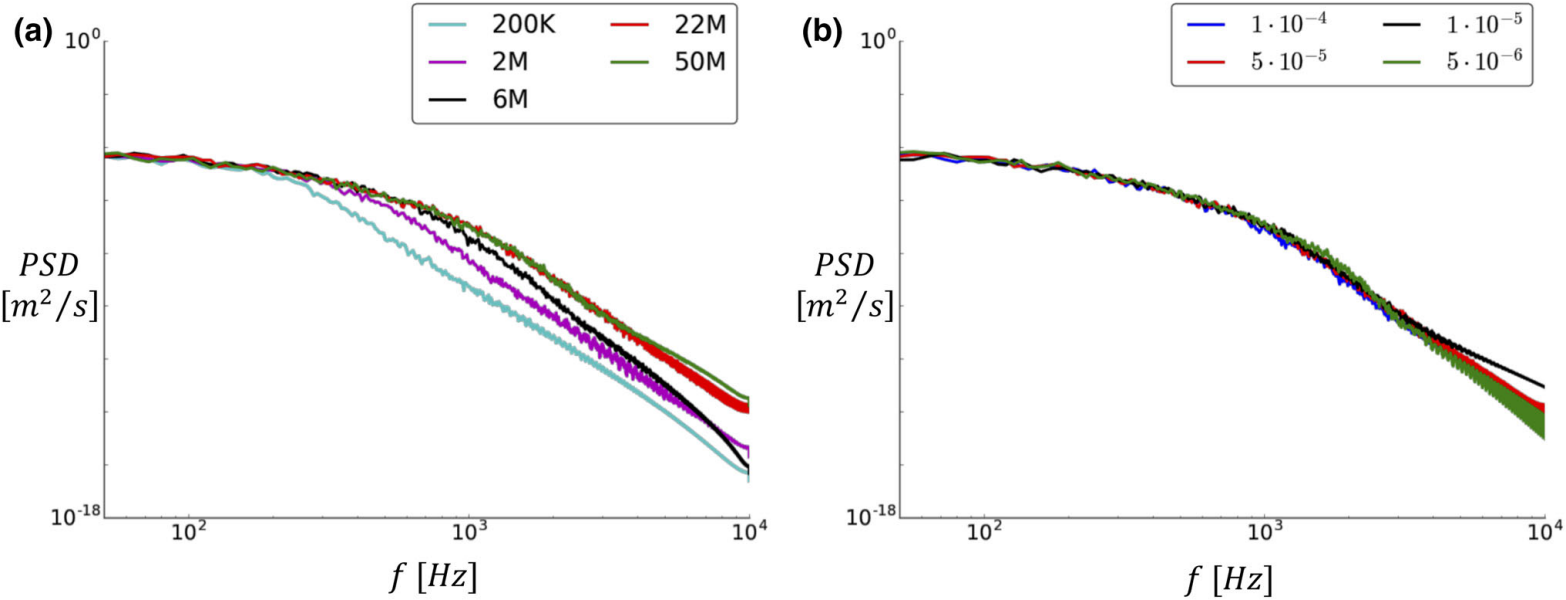
Power spectral density of the numerous meshes (a) and time steps (b) evaluated at point **P**.

We also investigated the power spectral density (PSD) of the magnitude of the fluctuating velocity ($\varvec{u^{\prime}}(x, t)|$) at point **P**. In Fig. 7a, it is possible to observe again that the 200K, 2M, and 6M simulations differed from the two finest simulations, which for all practical purposes were indistinguishable.

To further support these results, we also show the $tke$ for all available meshes in slices A–D (Fig. 12 in Appendix A) and the PSD of the fluctuating velocity, $\varvec{u^{\prime}}(\varvec{x},t)$, in five additional points along the ICA.

Based on these observations, the 22M simulation was considered to be the best tradeoff between computational cost and accuracy. The temporal refinement study was hence performed on the 22M mesh.

#### Temporal Refinement Study

The temporal refinement simulations were evaluated similarly to the spatial refinement study. In all lines, the $tke$ with $\Delta t$ equal to $5 \times 10^{ - 5}$, $1 \times 10^{ - 5}$, and $5 \times 10^{ - 6}$ (Fig. 6b) were close to indistinguishable. In contrast, the $1 \times 10^{ - 4}$ simulation differed slightly in the lines on slices A, B, and C. The contours for the available time steps are shown as well in the appendix A, with consistent results. Furthermore, the PSD of the fluctuating velocity compoent shown in Fig. 7b confirms that the impact of temporal refinement is small, however the plots in Figs. 17, 18, 19, 20, 21, and 22 of Appendix B show a slight difference between $\Delta t = 1 \times 10^{ - 4}$ and the rest.

Based on the results from the spatial and temporal refinement study the 22M mesh ($\Delta x = 1.92^{ - 4}$ m) and time step of $\Delta t = 5 \times 10^{ - 5}$ seconds offered the best tradeoff between computational cost and accuracy, and is now referred to as the *reference solution*.

### Large Eddy Simulations

#### Constant Flow Simulations

**Figure 8:**
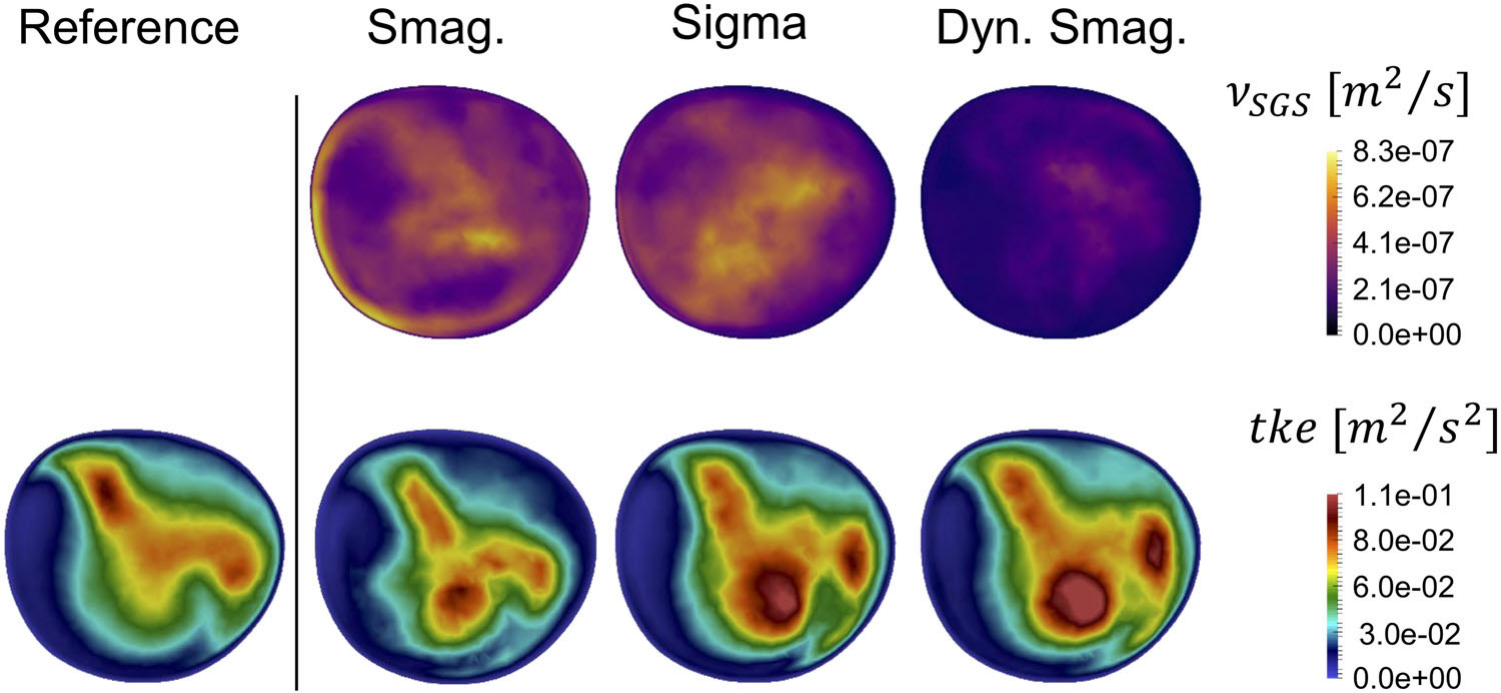
Time-averaged SGS viscosity $\nu_{SGS}$ (upper panel) and resulting turbulent kinetic energy (bottom panel) for the tested SGS models and compared with the reference solution at slice C. The Smagorinsky model is phenotypically different, both in $tke$ pattern and magnitude. Although more similar, the Sigma and Dynamic Smagorinsky did not produce the same $tke$ patterns.

For the constant flow rate LES simulations we first focused on the added viscosity from the SGS models ($\nu_{SGS}$), and $tke$ in slice C, as shown in Fig. 8. Although the order of magnitude of $\nu_{SGS}$ is the same for all models, the $tke$ of the Smagorinsky model was phenotypically different showing an improper near-wall behavior. Due to the shortcomings of the Smagorinsky SGS model, it was not included in the pulsatile flow simulation discussed further below. The added viscosity of the Dynamic Smagorinsky model was overall lower compared to the Sigma model, but leading to a comparable $tke$. Both models show a similar swirly pattern, and flow instabilities are clearly recognizable. However, the peak values in the bottom-center area of the slices highlighted that the location of the highest $tke$ intensities are shifted compared to the $tke$ of the reference simulation, showing that the LES flow simulations did not perfectly recreate the reference solution. For completeness, the $\nu_{SGS}$ and $tke$ in slices A–D are shown in Appendix A, Figs. 14a and 15a, respectively.

**Figure 9:**
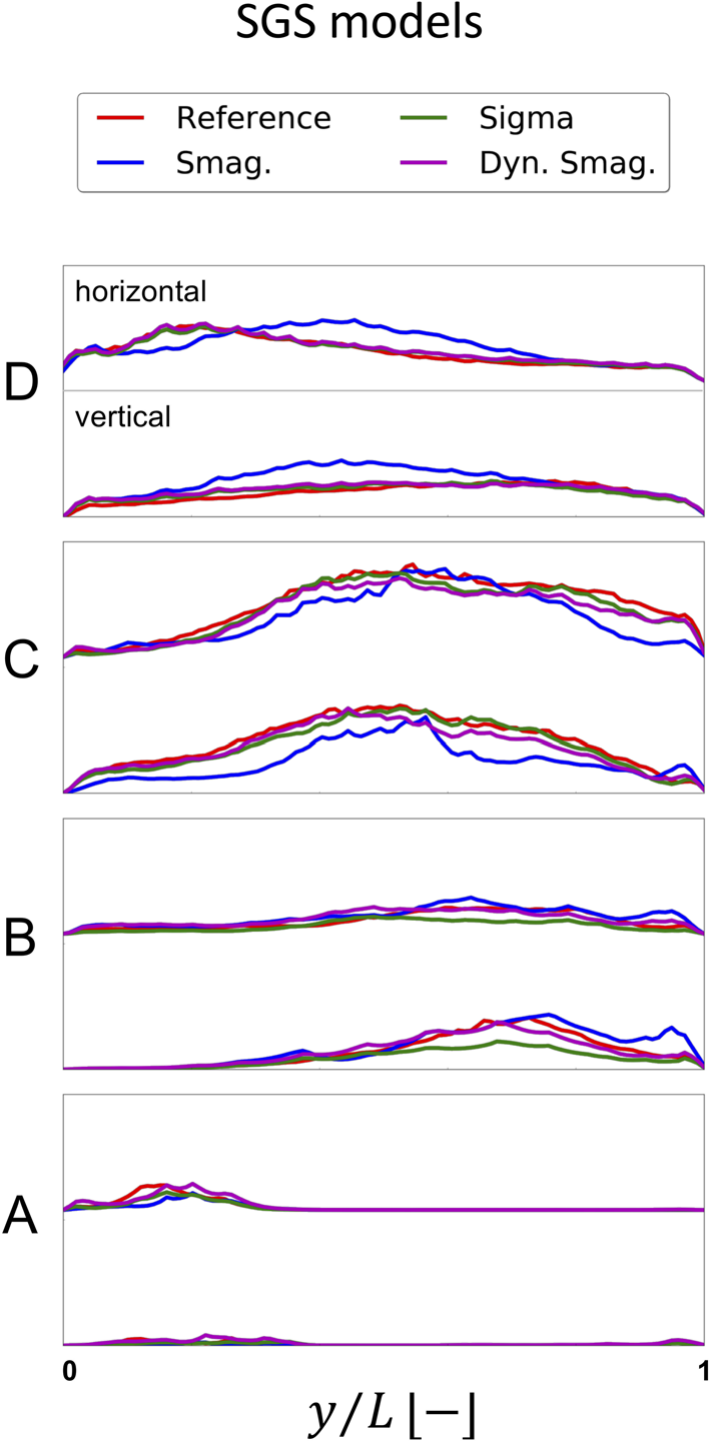
Time-averaged $tke$ over vertical and horizontal lines on slices A to D for the reference solution and the three SGS models (Smagorinsky, Sigma, Dynamic Smagorinsky).

For a further comparison of the SGS models with the reference solution, the $tke$ values along the horizontal and vertical line on the A–D slices was reported in Fig. 9. The Smagorinsky model did not produce a comparable $tke$ on any line. The Sigma and Dynamic Smagorinsky were able to replicate the reference solution on plane A and D but not on B and C, which are the ones more affected by the presence of a stenosis and hence the most relevant for the study of high-frequency flow instabilities. In the near-wall region of plane B, particularly, it is possible to appreciate the different properties of the SGS models. The $tke$ obtained with both Smagorinsky models is importantly affected by the $\nu_{SGS}$. The Sigma model is, on the other hand, far more dissipative, since the $D_{m}$ of the model proposed by Nicoud *et al.*^29^ would vanish in case of two-dimensional or two-components flow, and in case of pure rotation or pure shear, and would behave cubicly in the near-wall region.

**Figure 10:**
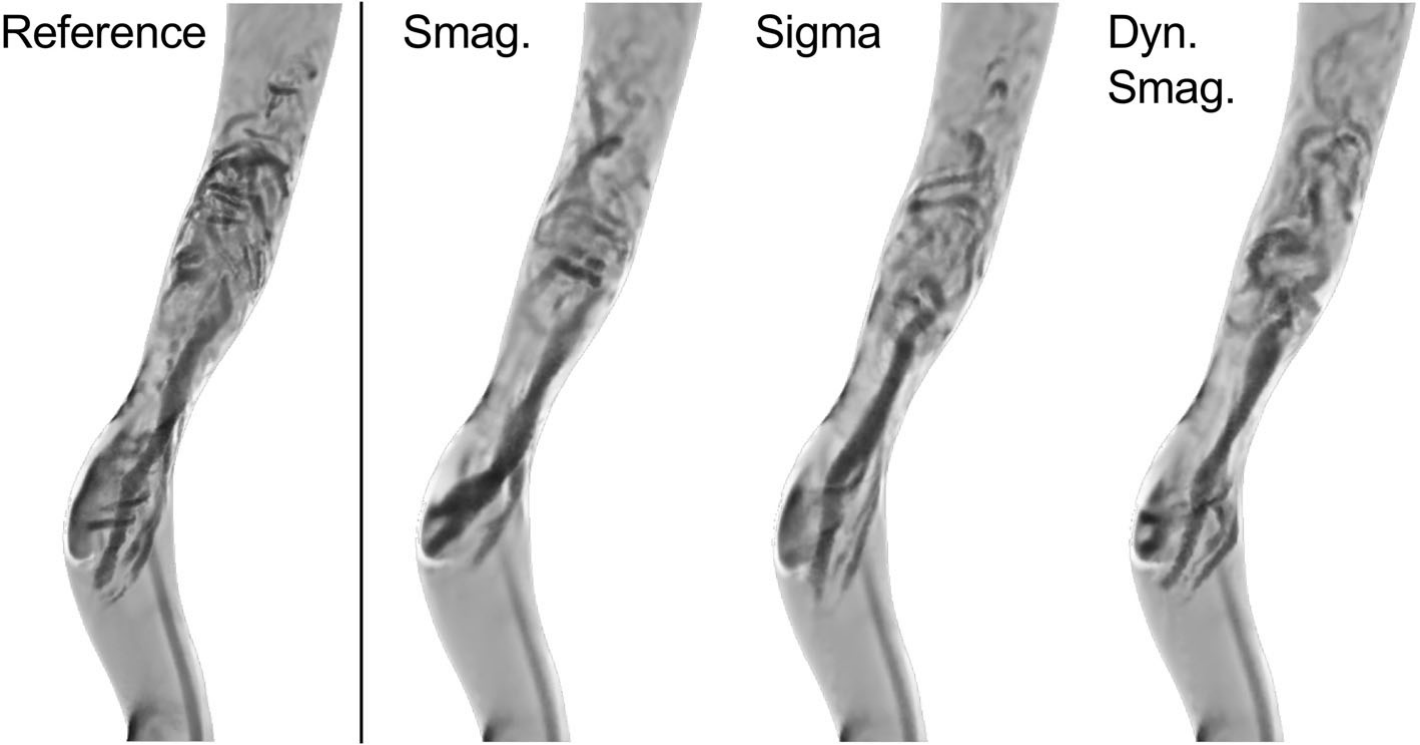
Vortexes in the ICA region was identified by means of the Q-criterion. The breakdown location of the jet and the intensity of flow instabilities of all LES simulations differed from the reference solution.

A visual inspection of the Q criterion (Fig. 10) confirmed that the post-stenotic jet did not break down as rapidly as the reference solution for any of the tested SGS models.

We also compared the computational cost (CPU hours) of the SGS models, the reference solution, and the 6M simulation without SGS models (Table 4). The reference solution (22M-None) was 4.60 times more computationally expensive compared to the 6M mesh simulation without any SGS model (6M-None), while the Dynamic Smagorinsky and the Sigma models were more expensive than the 6M-None. The comparison in computational cost between the Dynamic Smagorinsky and Sigma model is consistent with previous studies.^30^, ^33^

**Table 4:** CPU hours of the reference solution (22M-None) and of the most relevant SGS models compared to the 6M-None for constant flow rate.

Mesh	SGS model	CPU h/6M-None CPU h	s/time step
22M	None	4.60	2.43
6M	None	1.00	1.58
6M	Sigma	1.77	2.80
6M	Dynamic Smagorinsky	2.66	4.20

There is a marked difference between the 22M and the SGS models. The amount of time required to perform each time step $\Delta t$ highlights remarkable differences between Dynamic Smagorinsky SGS model and 6M-None

#### Pulsatile Flow Simulations

**Figure 11:**
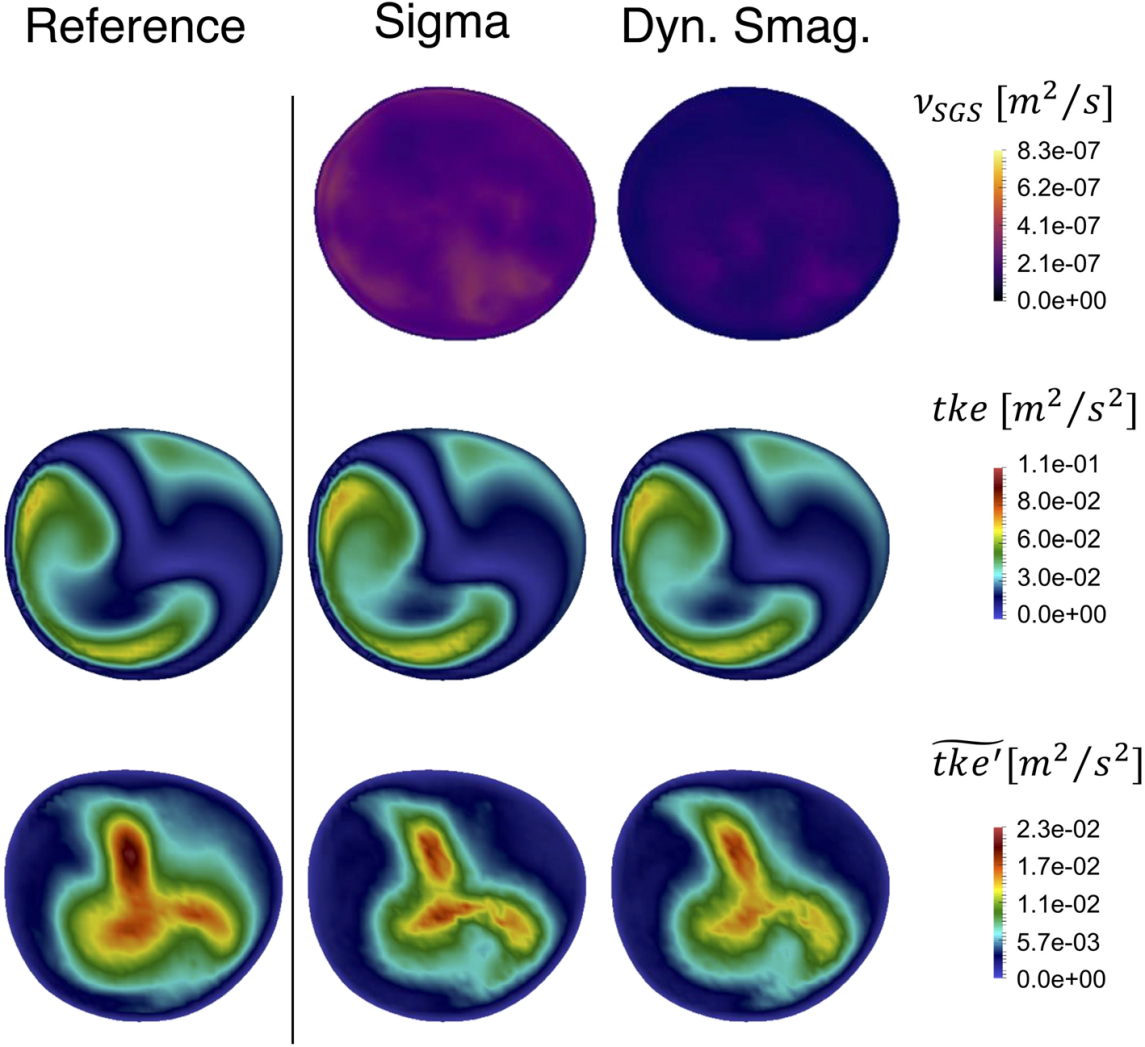
Time-averaged added SGS viscosity $\nu_{SGS}$ (upper panel), resulting time-averaged $tke$ (middle panel) and its high frequency counterpart $\widetilde{{tke^{\prime}}}$ (bottom panel) for the tested SGS models and compared with the pulsatile reference solution on slice C. Both $tke$ and $\widetilde{{tke^{\prime}}}$ are alike for the LES simulations and the reference solution.

**Figure 12:**
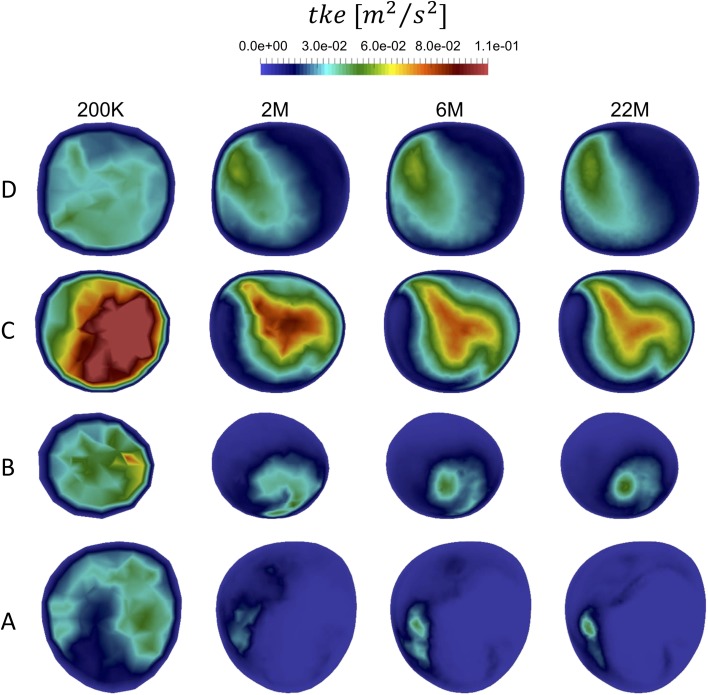
Comparison of several mesh densities, based on qualitative analysis of the time-averaged turbulent kinetic energy distribution over several slices (A, B, C, D) along the ICA.

The evaluation of the SGS models applied to a pulsatile inflow was performed with Sigma and Dynamic Smagorinsky models. The time-averaged SGS viscosity $\nu_{SGS}$ (Fig. 11—upper row) was globally higher for the Sigma compared to the Dynamic Smagorinsky model, consistent with the constant peak systolic flow rate simulations.

In the middle row of Fig. 11 we show $tke$ of the pulsatile simulations, where $\varvec{u}^{\varvec{'}} \left( {\varvec{x},t} \right)$ was computed with a constant $\bar{u}$, in slice C for the reference solution, the 6M with Sigma model, and 6M with Dynamic Smagorinsky model, left to right respectively. Visually, there was a large similarity between the $tke$ fields.

**Figure 14:**
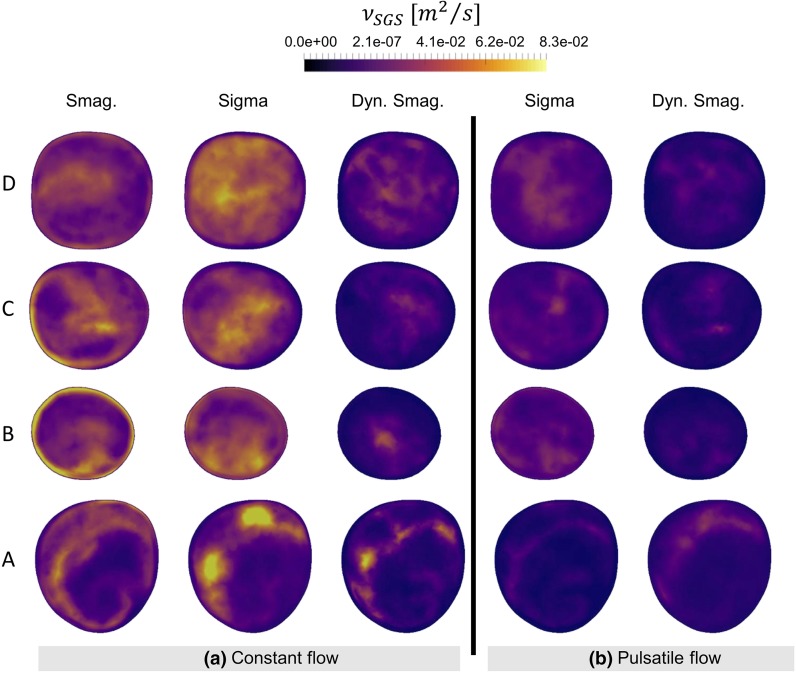
Comparison of sub-grid scale viscosity for the LES simulations used in this study on the 6M mesh with $\Delta t = 5 \times 10^{ - 5}$ seconds, with constant flow rate ($\left( {Re = 1380} \right)$ on the left hand side (a) and pulsatile flow rate $\left( {Re_{average} = 980, \;Re_{\hbox{max} } = 1380} \right)$ on the right hand side (b).

**Figure 15:**
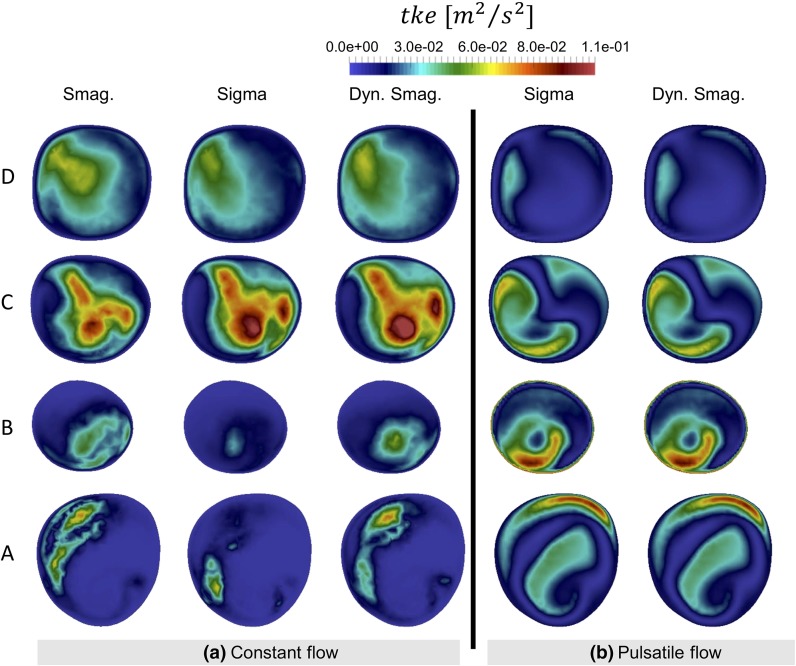
Comparison of the time-averaged turbulent kinetic energy field resulted from applying the sub-grid scale models in the LES simulations, for constant (a) and pulsatile (b) flow rate.

**Figure 16:**
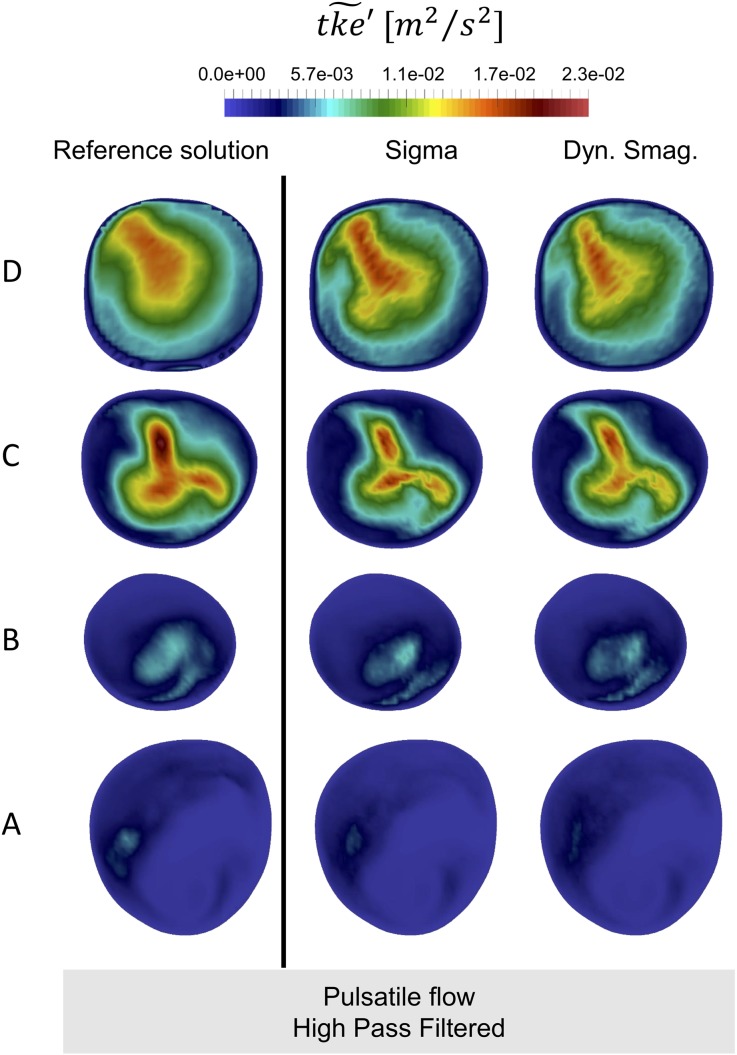
Comparison of the time-averaged high-pass filtered turbulent kinetic energy field for pulsatile simulations. The $\widetilde{{tke^{\prime}}}$ for the reference solution simulation (22M None) is reported on the left hand side, while the $\widetilde{{tke^{\prime}}}$ for the most relevant LES simulations (Sigma model and Dynamic Smagorinsky model) are depicted in the right hand side.

**Figure 17:**
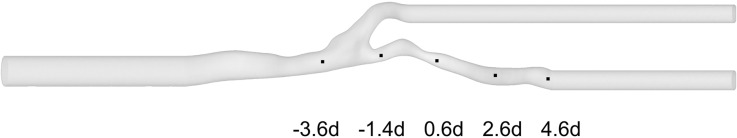
Centerline locations where the PSD of the velocity traces was computed and whose plots are reported in the following paragraphs of this Appendix.

**Figure 18:**
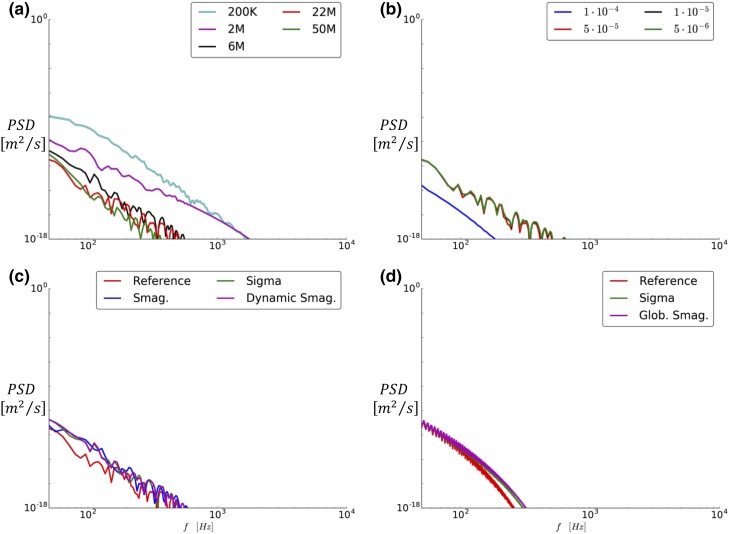
PSD of the velocity obtained by probing on a point located 3.6 diameters upstream of the stenosis on the centerline of the model, showing that the flow is laminar before the carotid bifurcation.

**Figure 19:**
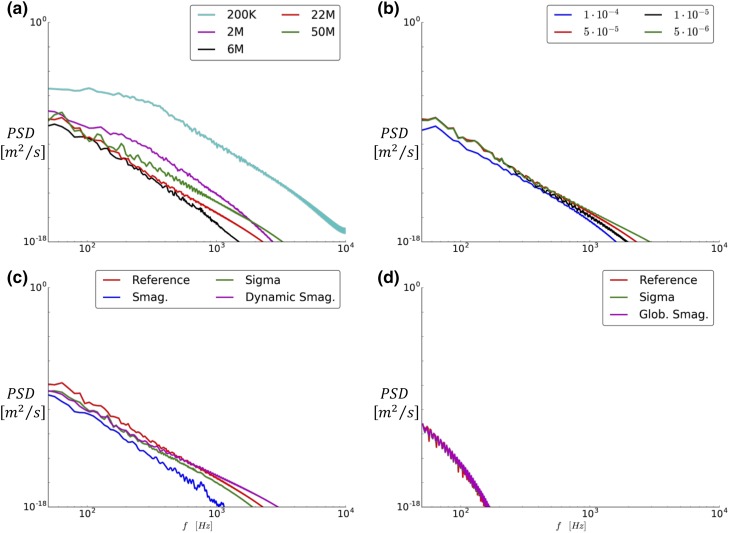
PSD of the velocity obtained by probing on a point located 1.4 diameters upstream of the stenosis on the centerline of the model, after the carotid bifurcation, but before the stenosis, showing that for the constant flow rate simulations the flow instabilities have grown.

**Figure 20:**
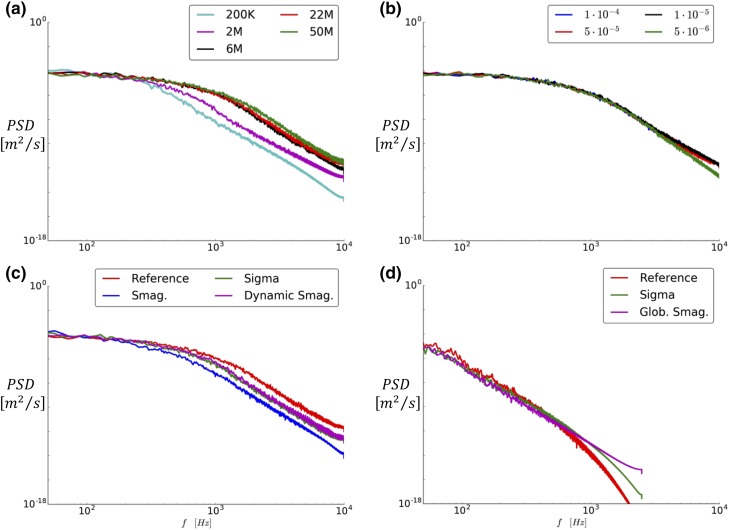
PSD of the velocity obtained by probing on a point located 0.6 diameters downstream of the stenosis on the centerline of the model, showing that the flow is highly unstable and that the jet has already broken down.

**Figure 21:**
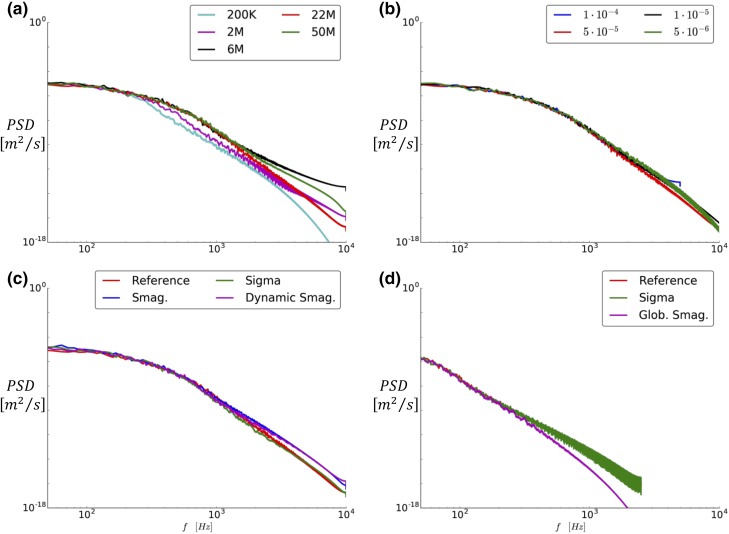
PSD of the velocity obtained by probing on a point located 2.6 diameters downstream of the stenosis on the centerline of the model, showing that the energy has decreased in the high-frequent region of the flow, and that the flow is starting to relaminarize.

**Figure 22:**
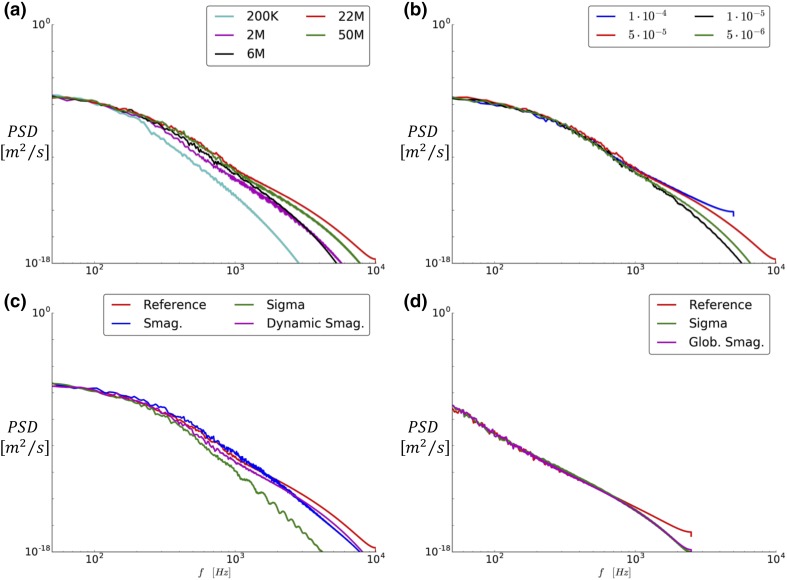
PSD of the velocity obtained by probing on a point located 4.6 diameters downstream of the stenosis on the centerline of the model.

The bottom row of Fig. 11 shows that the $tke$ computed by using high-pass filtered fluctuating velocity components, $\varvec{u'}(\varvec{x},t$), now referred to as $\widetilde{{tke^{\prime}}}$, is highly comparable. It is hence clear that the LES simulations harbor the same turbulent kinetic energy as the reference solution. The power spectral densities in Appendix B Figs. 18, 19, 20, 21 and 22, showing no difference in between the LES models and the reference solution, also backed up these results. Of note are also the large differences between the two measures of $tke$ in the pulsatile simulation. The bottom row represents the regions with turbulent kinetic energy with a higher frequency fluctuation than those introduced from the flow waveform at the inlet. In the context of this study, we emphasize these more as the high frequency content of the simulated flow is our quantity of interest. For completeness, the $\nu_{SGS}$, the $tke$ and the $\widetilde{{tke^{\prime}}}$ in slices A–D are shown in Appendix A, Figs. 14b, 15b and 16, respectively.

Table 5 shows CPU hours computed for the two SGS models, the reference solution, and the 6M mesh without any SGS model applied (6M-None) for pulsatile flow simulations. Similar to the constant flow rate simulations, the reference solution (22M-None) was more computationally expensive than any other 6M simulation, and the LES simulation run with the Dynamic Smagorinsky model was again more expensive than the 6M-None and 6M-Sigma simulations.

**Table 5:** CPU hours of the reference solution (22M-None) and of the most relevant SGS models compared to the 6M-None for pulsatile flow rate.

Mesh	SGS model	CPU h/6M-None CPU h	s/time step
22M	None	4.18	3.65
6M	None	1.00	2.62
6M	Sigma	1.49	3.90
6M	Dynamic Smagorinsky	2.53	6.64

Since the computational results of the two SGS models were comparable to the reference solution, the reduction in computational cost favored the Sigma model.

## Discussion

The aim of this study on post-stenotic flow instabilities in a patient-specific model of a stenosed carotid artery, was two-fold: (1) find an adequate under-resolved DNS solution from a spatial and temporal refinement study with respect to the turbulent scales, and from a pragmatic biomedical engineering point of view, and (2) assess whether three commonly used SGS models are able to replicate the results from our reference solution for both constant and pulsatile flow rates on a coarser mesh (Table 6).

**Table 6:** Space and cycle-averaged velocity compared and extrapolated with Richardson’s extrapolation method for different grid sizes.

Number of elements	$\bar{u}$ (m/s)	*A* (m^2^)	*f* (m^3^/3)	% Error
6M	$2.2265 \times 10^{ - 1}$	$1.8289 \times 10^{ - 4}$	$4.1426 \times 10^{ - 4}$	8.19
22M	$2.3295 \times 10^{ - 1}$	$1.7165 \times 10^{ - 4}$	$3.9989 \times 10^{ - 4}$	4.44
50M	$2.3636 \times 10^{ - 1}$	$1.6691 \times 10^{ - 4}$	$3.9453 \times 10^{ - 4}$	3.04
Richardson extrapolation	–	–	$3.8290 \times 10^{ - 4}$	0.00

Focusing firstly on the spatial and temporal refinement studies, we found a grid spacing of $\Delta x = 1.92 \times 10^{ - 4} \; {\text{m}}$ and time step size of $\Delta t = 5 \times 10^{ - 5} \;{\text{s}}$ to be the best tradeoff between computational cost and accuracy from a pragmatic biomedical engineering point of view.

Relative to others, Lancellotti *et al*.^18^ reported using $\Delta t = 6.25 \times 10^{ - 4}$ and an “effective” mesh size of $\Delta x = 6.5 \times 10^{ - 5}$ as a reference solution, i.e., a time step one order of magnitude larger, and a cell size almost three times smaller. Even with a different working fluid, their Reynolds number was only 25% smaller at the stenosis. Lee *et al*., which was the first to performed true DNS in a patient-specific geometry, is not directly comparable with respect to mesh size since they used spectral finite elements, but they reported a time step of $\Delta t = 1 \times 10^{ - 5}$, although argued from a numerical stability point of view.^19^

Furthermore, we found that simulations on a coarser mesh with SGS models were not able to capture the high-frequency flow features for a constant flow rate equivalent to the peak systolic flow rate ($Re = 1380$). If pulsatile flow was applied ($Re_{average} = 980, Re_{\hbox{max} } = 1380$) the SGS models were able to replicate the flow features of the reference solution. The SGS models are thus applicable for investigating the turbulent-like flow in multiple patients or configurations, and ultimately, with fluid structure interaction (FSI), how the flow fluctuations can present as skin vibrations on the neck of affected patients. The discrepancies between constant and pulsatile flow simulations can be attributed to the differences in flow rate, which for the constant flow rate simulation, was higher than the average flow rate for the pulsatile flow rate simulations, although with the same peak flow.

The SGS viscosity of the Sigma model was overall higher than the one resulting from the application of the Dynamic Smagorinsky model, which is the opposite of what Baya Toda *et al*.^2^ found in their study of an internal combustion (IC) engine. In their IC chamber, the viscosity of the Dynamic Smagorinsky model increased when the flow jet impinged a wall, as a consequence of the increase of the strain-rate tensor. On the contrary, the Sigma model did not produce an increased SGS viscosity in that region, as its $D_{m}$ is not affected by the magnitude of the strain-rate tensor. The different dissipation provided by the two model was, therefore, a direct consequence of the physics of an impinging jet, which are not comparable to the physics of a free jet such as the one considered in this manuscript. We therefore recommend a careful evaluation of the choice of SGS model with respect to the physical problem, keeping in mind that there is a sensitivity which can affect the results.

In our temporal refinement study the most accurate simulation had a time step size one order of magnitude smaller than our reference solution, and we can therefore, with great confidence, say that it was temporally well resolved. However, for the spatial refinement the averaged cell lengths in the finest mesh was only 33% smaller compared to our reference solution, see Table 1. To further investigate whether the finest mesh could be considered a valid point of reference for the spatial sensitivity study, we used Richardson’s extrapolation method^47^ defined as:

4 $$f_{extrapol. } = f_{{\Delta x_{i} }} + c \times \Delta x_{i}^{p} ,$$

where $f_{{\Delta x_{i} }}$ is the quantity of interest, here a temporal and spatial average of $\varvec{u}(\varvec{x}, t)$, $\Delta x_{i}$ is a measure of grid spacing, *c* is a constant value obtained from the evaluation of Eq. (4) $f_{{\Delta x_{i} }}$ at the two finest meshes (22M and 50M), *p* is the measured order of convergence for $f_{{\Delta x_{i} }}$, and subscript *i* is mesh number in the refinement. In order to nullify the impact of difference in inlet size we normalized $\bar{u}$ by the inlet cross sectional area $A_{i}$ of each mesh (which is different due to different accuracy with which the boundary of the inlet can be replicated). The results of the Richardson’s extrapolation are summarized in Table 5. The solution was monotonically converging across the three finest grids with an obtained order of convergence $p = 1.34$. Furthermore, the uncertainties on the finest grid and on the second finest grid were not greater than 10% even with a security factor of 1.5,^6^ and could therefore be considered converged with respect to the mean flow features.

Although the mean flow was converged, the question remains if the flow was well resolved at the smallest scales. We previously discussed this in Mancini *et al*.,^24^ where we computed the Kolmogorov length scale (*μ*).^17^ We computed the ratio of the local cell length, $\Delta x$, and the Kolmogorov length scale, *μ*, in each cell, and reported the temporal and spatial global maximum. The ratio on the two finest meshes were below 10, typically sufficient to capture > 95% of the dissipation.^31^ However, considering the temporal averaged ratio in the post-stenotic region, we obtained a mean/max of 0.347/1.11, and 0.463/1.755 for the 22M and 50M simulation, respectively, showing that the flow is well-resolved. We also obtained equal results from computing $l^{ + }$, a surrogate measure for the Kolmogorov length scale compared to the local cell length,^43^ with $l_{\hbox{max} }^{ + } = 6.2$ located along the wall in the stenosis. Performing a true DNS simulation would require building a mesh with cell lengths of roughly seven times smaller than the finest mesh, yielding mesh consisting of roughly 21 billion cells and thus requiring an enormous amount of computational resources while, from a pragmatic biomedical engineering point of view, having no added value. That being said, using an adaptive mesh strategy would yield a DNS simulation with a smaller mesh, however our local refinement approach is simple to adapt, and the local refinement is consistent between spatial refinement levels. It is also noteworthy that the homogeneous isotropic assumption of the Kolmogorov hypothesis was not met in our simulations. The Kolmogorov hypothesis therefore underestimates the smallest scales, and the simulation might therefore be even more well resolved than the Kolmogorov length scale indicates.

Based on our numerical investigations, we have a high level of confidence in our numerical results, but how do the results compare to flow *in vivo*? *Firstly*, we assumed a Newtonian fluid with properties mimicking water. With realistic flow rates, the Reynolds number was hence 3.3 times larger compared to *in vivo *blood flow, since blood has a higher kinematic viscosity compared to water. As a result, the intensity of the post-stenotic turbulent-like flow is higher in our numerical experiments than what can be expected *in vivo*. The spatial and temporal resolutions of this study still hold for physiological realistic situations, in terms of being well-resolved. On the other hand, the use of water also allows for valuable comparison with *in vitro* experiments, in which water is often used instead of glycerin-based blood-mimicking fluids. Moreover, the relative effect of assuming a non-Newtonian fluid has shown to be small in the carotid bifurcation since, due to the high share rates in the carotid bifurcation, the non-Newtonian models work in a regime where the viscosity is close to a constant value.^20^ Furthermore, compared to other uncertainties like modeling choices and segmentation, the assumption of a Newtonian fluid is marginal. Moreover, from a physical point of view, blood is more complex than just being a non-Newtonian fluid, it is also multiphase flow. The presence of small particles, to mimic red blood cells, has been found to dampen flow instabilities,^1^ but should not phenotypically change the flow.

*Secondly*, we modeled the walls as rigid, but they are naturally compliant. Modeling the stenosis with compliant models might dampen some of the unstable flow features. However, determining the material properties of the stenosed region are therefore challenging, since it consists of both plaque and lipid pools. The former is stiffer than a healthy vessel wall, while the latter is more compliant. Therefore, anticipating how this might affect the results are challenging. Moreover, the soft tissue embedding the carotid arteries would further dampen the instabilities that would present on the skin surface, generally with a lower amplitude relative to the fluctuations induced by the turbulent-like post-stenotic flow.^5^

In total, the simulation results cannot directly be translated into an *in vivo *situation, but is an important first step towards a trustworthy patient-specific flow simulation for investigating the high-frequent flow fluctuations. Future efforts will be directed towards validating the flow with *in vitro* experiments, and using an FSI approach, i.e., incorporating the effect of compliant walls, which is well-known to better mimic physiological conditions.^40^

To ease comparison with other solvers, increase reproducibility, and promote openness in science, we also provide an additional repository^25^ with our problem file for the Oasis solver, the used meshes, and averaged results from the reference solution with constant flow rate. The results from this study can therefore easily be compared to other solvers, and potentially ease the amount of work needed to show that a given solution strategy is adequate for investigating turbulent-like flow features for a post-stenotic flow.

In our line of research we are interested in the high-frequent flow features, however a plurality of studies using CFD to investigate flow in the carotid bifurcation are interested in (time-averaged) wall share stress (WSS), or other WSS-derived quantities.^4^, ^34^, ^46^ We have not investigated whether a coarser mesh would be sufficient for investigating WSS, and furthermore, how LES models would affect the results. For comparison, Valen-Sendstad *et al*. found that time-averaged wall shear stress (WSS) was relatively insensitive to the applied computational solution strategy, whereas the OSI, more sensitive to flow fluctuations, changed significantly.^46^ We therefore caution readers about extrapolating our results to studies investigating WSS-derived quantities.

Lancellotti *et al*.^18^ investigated the applicability of LES models in pulsatile simulations of a stenosed patient-specific carotid bifurcation. They, like us, found a static Sigma model to perform best. Although we agree with most of their conclusions, there are some caveats of the study, e.g., they used a streamline upwind/Petrov–Galerkin pressure stabilized Petrov–Galerkin formulation for the reference solution, known to add numerical diffusion,^41^ which would not be equal between the two meshes. Despite these limitations, we can conclude that both Lancellotti *et al*.^18^ and the current study could be used as points of reference for LES modeling and spatial and temporal refinement when investigating flow in the carotid bifurcation. In particular, Lancellotti *et al*. for WSS and the current study for the high frequent flow features.

We can thus state that future studies investigating the high-frequent features of post-stenotic flow can use an equivalent spatial and temporal resolution reported here, with a Sigma SGS model. Of note is that if a higher Reynolds number is applied, like in the constant flow rate simulations presented here, the turbulence models were not applicable at reported spatial resolution. More specifically, we will use these results to investigate the possibility to diagnose carotid stenosis based on the amplitude of the unstable flow’s frequency content by measuring the neck skin vibrations.

## Conclusions

The numerical methodology applied in this study allowed us to properly resolve the flow field of a stenosed patient-specific carotid bifurcation and hence detect instabilities induced by the stenosis. When compared to the *reference solution*, only Sigma and Dynamic Smagorinsky were able to replicate the averaged mean flow features from the constant flow rate simulation, and the turbulent flow features in the pulsatile flow rate simulations. The computational cost was lower for the Sigma model, and therefore the best choice balancing accuracy with computational cost for studying high frequency flow instabilities. However, for higher Reynolds numbers, similar to the constant flow rate simulation, the LES models were not sufficient. Future efforts on this subject should be conducted with the abovementioned SGS model, while taking advantage of a robust high-order numerical solver such as the one used in this study.

## Figures and Tables

**Figure 13:**
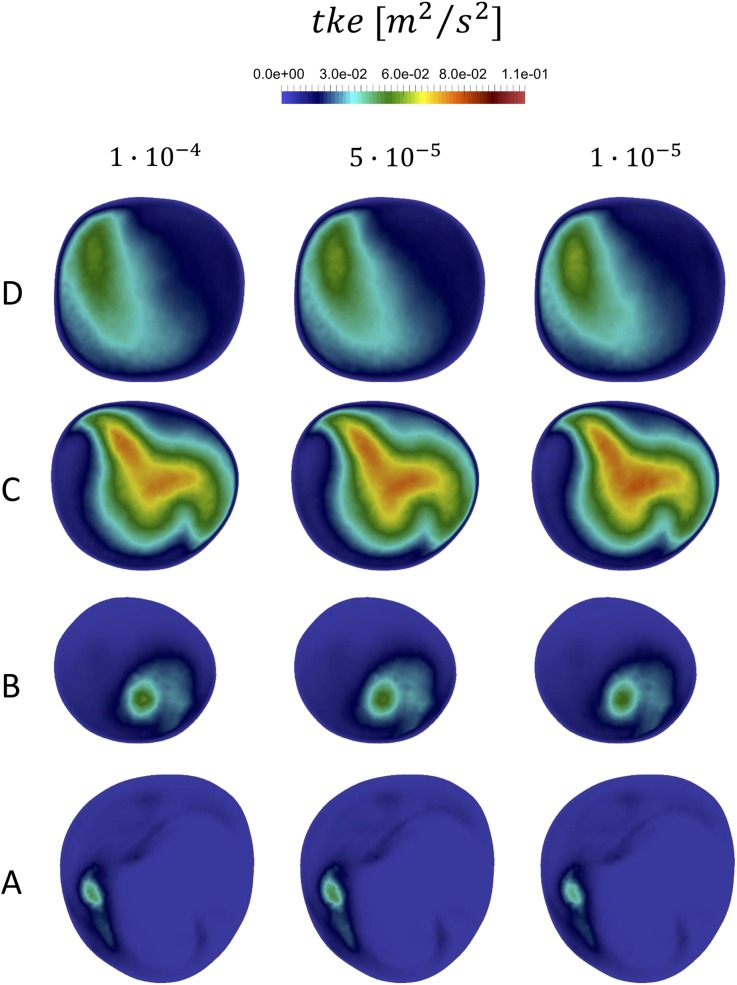
Comparison of the time-averaged turbulent kinetic energy field on the depicted slices for several temporal resolutions used in this study.

## Appendix A

In this section, the time-average of the most relevant parameters, i.e. turbulent kinetic energy ($$tke$$), subgrid-scale viscosity ($$\nu_{SGS}$$) and high-pass filtered $$tke$$$$\left( {\widetilde{{tke^{\prime}}}} \right)$$ if applicable, are depicted for each simulation on the slices of interests (A to D). Specifically, $$tke$$ is reported for the mesh and temporal sensitivity analysis in Figs. 12 and 13, respectively, while $$\nu_{SGS}$$, $$tke$$ and $$\widetilde{{tke^{\prime}}}$$ are shown in Figs. 14, 15, 16 for the LES simulations.

### Mesh Sensitivity Analysis

Time-averaged turbulent kinetic energy ($tke$) contours for the available meshes (Fig. 12). The contours were not produced for the finest mesh because the expected redundancy did not justify the extremely high investment in computational resources. These simulations were run with a time step of $\Delta t = 5 \times 10^{ - 5}$ seconds, without any SGS model.

### Time Step Sensitivity Analysis

Time-averaged $tke$ contours for simulated time steps on the 22M mesh (Fig. 13). The contours for the finest time step $\left( {\Delta t = 5 \times 10^{ - 6} \;{\text{s}}} \right)$ were not produced because the expected redundancy did not compensate the investment of the several CPU hours required.

### Sub-Grid Scale Models

Time-averaged sub-grid scale viscosity ($\nu_{SGS}$) for the three tested SGS models with constant and pulsatile flow (Fig. 14). These simulations were run on the 6M mesh and $\Delta t = 5 \times 10^{ - 5}$ s.

Resulting time-averaged $tke$ fields for constant, pulsatile flow rates and pulsatile flow rates (Fig. 15).

Resulting time-averaged turbulent kinetic energy fields for constant, pulsatile flow rates and pulsatile flow rates with high pass filter applied ($\widetilde{{tke^{\prime}}}$) (Fig. 16).

## Appendix B

In this section, the power spectral densities (PSD) at several centerline points in our model (see Fig. 17) are depicted in Figs. 18, 19, 20, 21 and 22 for spatial (a) and temporal refinement studies (b), for LES simulations with constant (c) and pulsatile (d) flow rate.
